# Goat Milk-Derived Extracellular Vesicles Alleviate Colitis Potentially Through Improved Gut Microbiota in Mice

**DOI:** 10.3390/foods14091514

**Published:** 2025-04-26

**Authors:** Xinru Wang, Yi Liu, Hong Chang, Hein-Min Tun, Xiaodong Xia, Ye Peng, Ningbo Qin

**Affiliations:** 1 SKL of Marine Food Processing & Safety Control, National Engineering Research Center of Seafood, School of Food Science and Technology, Dalian Polytechnic University, Dalian 116034, China; 2 Jockey Club School of Public Health and Primary Care, Faculty of Medicine, The Chinese University of Hong Kong, Hong Kong SAR 999077, China; 3 Microbiota I-Center (MagIC), Hong Kong SAR 999077, China; 4 Li Ka Shing Institute of Health Sciences, Faculty of Medicine, The Chinese University of Hong Kong, Hong Kong SAR 999077, China

**Keywords:** goat milk, extracellular vesicles, ulcerative colitis, gut microbiota, mouse model

## Abstract

Ulcerative colitis (UC) is characterized clinically by intestinal inflammation and gut microbiota dysbiosis. The consumption of biologics, although effective in inflammation control, may lead to adverse effects and is inconvenient for at-home administration. Goat milk-derived extracellular vesicles (GMEVs) have been proposed as a supplement to prevent intestinal inflammation. However, their therapeutic potential for colitis remains elusive. This study aimed to explore the preventive effect of GMEVs on colitis and its underlying mechanisms through the microbiota-immune axis using a dextran sodium sulfate (DSS)-induced colitis mouse model. We found that a pre-treatment of 20 mg/kg/d GMEVs effectively prevented body weight loss, colon shortening, the depletion of colonic goblet cells, and the disappearance of crypts, while enhancing the intestinal mucosal barrier. Consistent with these phenotypes, GMEV pre-treatment increased levels of IL-22 and IL-10 and decreased levels of IL-1β, TNF-α, IL-6, and iNOS. However, GMEVs themselves had no effect on normal mice. Paralleling the alleviation of intestinal inflammation, GMEV pre-treatment also restored the reduction in *unclassified Muribaculaceae*, *Dubosiella*, and *Lactobacillus* and suppressed the expansion of *Alistipes* and *Proteobacteria* following DSS treatment. Additionally, GMEV intake significantly downregulated the expression of proteins in the NF-κB signaling pathway induced by DSS. In summary, GMEVs could prevent colitis by regulating intestinal inflammation, the intestinal mucosal barrier, gut microbiota, organ damage, and the immune microenvironment. This study demonstrated that GMEVs have potential application prospects for UC prevention.

## 1. Introduction

Ulcerative colitis (UC) is a chronic inflammatory disease that directly affects the rectum and colon. The global incidence rate of UC is increasing, affecting approximately 5 million of the world’s population in 2023 [1]. The severity of UC is strongly related to a number of factors, including gut epithelial barrier defects, gut microbiota dysbiosis, and a dysregulated immune response [2]. Medications with different mechanisms, such as 5-aminosalicylic acid, steroids, immunosuppressants, and anti-TNF-α drugs, have been licensed to improve UC [3]. However, recent studies have reported serious side effects, such as toxicity and resistance, raising concern about the long-term applications of these drugs [4,5,6,7]. Thus, novel therapies and pharmacological substances for preventing UC are in high demand.

The gut is vitally essential in the immune system of the body. Apart from absorbing nutrients, the gut can also promptly respond to external harmful stimuli and pathogenic microorganisms [8,9]. The early weaning of neonatal animals leads to poor gut development, increases vulnerability to pathogens, and consequently triggers intestinal inflammation [10,11]. This is attributed to the early cessation of breast milk, which is rich in immunocompetent cells, miRNAs, proteins, and lipids. These immunomodulatory constituents provide immunity for newborns in order to prevent infections and promote immune system development [12,13]. The lipids, microRNAs, and proteins can be encapsulated in lipid bilayer nanovesicles called extracellular vesicles (EVs). EVs are secreted by cells into the surrounding environment, are responsible for cellular communication, and are linked to intestinal immune response and barrier function maintenance [14]. For example, Heidari et al. reported that adipose mesenchymal stem cell-derived EVs could relieve acute colitis induced by dextran sodium sulfate (DSS) via adjusting the Treg cell population and reducing the release of pro-inflammatory cytokines [15]. Despite the promising potentials, the natural secretion of EVs by human and animal cells is limited in quantities [16]. In contrast, foods and plants are recognized as appealing alternative sources of EVs in terms of biocompatibility, cross-species tolerance, cost-effectiveness, and availability [17].

Recently, immunoregulatory EVs have been documented in milk from various mammals, including humans, pandas, cows, pigs, and rodents [18]. For instance, EVs derived from human milk were shown to inhibit stimulated monocytes from secreting pro-inflammatory cytokines (IFN-γ, IL-2, and TNF-α) [19]. EVs derived from porcine milk can protect intestinal epithelial cells from apoptosis [20]. EVs derived from goat milk were liberated via the endosomal pathway and direct budding from the plasma membrane of mammary epithelial cells [21,22,23]. These goat milk-derived EVs (GMEVs) exhibit a protein composition closely resembling that of human milk, with significantly lower allergenicity compared to milk. GMEVs are enriched with bioactive components such as lactoferrin, milk fat globule membrane (MFGM) functional proteins (e.g., lectins and complement components), and epidermal growth factor (EGF), which collectively regulate gut microbiota and alleviate inflammatory responses. Furthermore, GMEVs carry multiple highly abundant conserved miRNAs (e.g., miR-148a, miR-26a, and let-7b), playing critical roles in anti-inflammatory processes and immune regulation [24,25]. GMEVs have been successfully used for drug delivery to diverse organs and for treating diseases [26]. Yenuganti et al. emphasized the effective antiviral activity of GMEVs against dengue virus [27]. Franzoni et al. showed that GMEVs polarized pig macrophages into an M1-like phenotype, which contributed to strengthening resistance to intracellular pathogens and malignant tumors [28]. In addition, Gao et al. proved that microRNAs in GMEVs alleviated LPS-induced intestinal inflammation [25]. Although GMEVs have received much attention for their prospective therapeutic and diagnostic applications, their effects on colitis have not been well studied.

The present study aims to explore the impacts of GMEVs on DSS-induced colitis in mice and elucidate the underlying anti-inflammatory mechanisms of GMEVs. The results could provide a theoretical foundation for developing breast-milk-based alternative therapeutics and their potential for improving the intestinal fitness of human beings and other mammals.

## 2. Materials and Methods

### 2.1. Preparation of GMEVs

GMEVs were extracted and purified using the continuous differential centrifugation method with optimization [25,29]. In brief, goat milk was centrifuged at 12,000× *g* for 30 min at 4 °C to skim fat. Following fat removal by centrifugation, the skimmed goat milk suspension was adapted to pH 4.1 with 2 M hydrochloric acid to precipitate the casein and then was centrifuged at 12,000× *g*. The supernatant was filtered via 0.22 μm filter membranes, and the filtrate was centrifuged at 130,000× *g* for 90 min at 4 °C with a CP 100 NX ultracentrifuge machine (Hitachi Co., Tokyo, Japan). Finally, the sediment was resolved in phosphate buffer solution (PBS, Solarbio, Beijing, China) to harvest GMEVs. EVs were kept at −80 °C before application.

### 2.2. Characterization of GMEVs

Protein contents of GMEVs were assayed by BCA kit (Solarbio, Beijing, China), and expressions of marker proteins (CD63 and TSG101) were determined by Western blot (WB). Vesicle diluent was dripped onto an electron microscope stage, sprayed with gold in a vacuum at an ultra-low temperature, and scanned by SU8010 SEM (Hitachi Co., Tokyo, Japan). An aliquot of 10 μL of vesicle diluent was dropped on a copper net to dry, and 2% phosphotungstic acid (Solarbio, Beijing, China) was applied to the copper net. Blends were dried at 25 °C for 6 min and observed by HT-7700 TEM (Hitachi Co., Tokyo, Japan) at 80 kV.

Another 10 μL of vesicle diluent was dripped onto a mica surface, dried naturally, and then placed on the sample table to scan the three-dimensional structure of the GMEVs with an AFM5500 AFM (Hitachi Co., Tokyo, Japan). The particle size distribution of the GMEVs was characterized using DLS (Brookhaven Instruments Co., New York, NY, USA) with a 90° scattering angle and 60 s duration at 25 °C. The particle concentration and zeta potential of the GMEVs were analyzed by ZetaView PMX120 Z (NTA, Particle Metrix, Meerbush, Germany) and Zetasizer Lab (Malvern Panalytical, Worcestershire, UK), respectively.

### 2.3. In Vitro Digestion Assay of GMEVs

An in vitro digestion experiment was conducted to evaluate the stability of the GMEVs. Artificial saliva, gastric juice, and intestinal fluid were purchased from Leagene Biotechnology Co., Ltd. (Leagene, Beijing, China). A total of 1 mL of GMEVs was combined with 1 mL of artificial saliva in a tube and incubated for 5 min, and an appropriate amount of the sample was taken for particle size detection. Then, 1.5 mL of the mixed solution after saliva digestion was sucked, mixed, and incubated with 1.5 mL of artificial gastric juice for 120 min. Subsequently, 2 mL of the mixed solution after gastric juice digestion was taken, mixed, and incubated with an equal volume of artificial intestinal fluid for 120 min to observe the performance of the GMEV particle sizes in the intestine. All incubations were carried out on a shaker at 37 °C.

### 2.4. Animal Experiments

Male C57BL/6J specific pathogen-free mice (7–8 weeks) were purchased from Liaoning Changsheng Biotechnology Co., (Benxi, China) (certificate number: DLPU2024043). All animal experiments were approved by Dalian Polytechnic University’s Animal Ethics Committee (approval number: DLPU2022078). Forty-two C57BL/6J mice were housed at 20 ± 2 °C under 60 ± 5% humidity and a 12 h light–dark cycle. After 7 days of adaptive feeding, the mice were separated into four groups, including the control group, the DSS group (3% DSS), the DSS + GMEVs group (3% DSS + 20 mg/kg/d GMEVs), and the GMEVs group (20 mg/kg/d GMEVs). Based on previous preliminary experiments showing that 20 mg/kg/d of GMEVs had the best intervention effect, the GMEVs group and DSS + GMEVs group were treated with 20 mg/kg/d GMEVs for the follow-up experiments, while the control group and DSS group were provided with equal volumes of PBS. After 14 days, 3% DSS was used to induce colitis in the DSS and DSS + GMEVs groups. Seven days later, blood, viscera, colon contents, and colon tissues of the mice were obtained for the following analyses.

### 2.5. Measurement of Serum Cytokines

Serum was separated from blood by centrifuging at 4000× *g* for 10 min at 25 °C. The levels of TNF-α, IL-1β, and IL-22 in the serum were then assayed by ELISA kits (Enzyme-linked Biotechnology Co., Shanghai, China). The absorbance of each sample was determined using an Infinite M200 at 450 nm.

### 2.6. Western Bolt Analysis

RIPA lysis buffer was used to extract total proteins in colon tissues and GMEV supernatant (Beyotime Biotechnology, Shanghai, China). The proteins of the GMEVs were separated by a special lysate for exosomes (Umibio, Shanghai, China). The protein concentrations of the samples were assayed by a BCA kit (Solarbio, Beijing, China). Subsequently, 5×SDS-PAGE loading buffer (Beyotime Biotechnology, Shanghai, China) was applied to the supernatant of the mixture and boiled in water for 10 min at 100 °C. The proteins were separated by 10% SDS-PAGE and subsequently electro-transferred to PVDF membranes. Following this step, the membranes were blocked with 5% skim milk powder for 1 h and then washed with TBST comprising 0.05% Tween-20 three times, each for 8 min. The protein bands were incubated with primary antibodies at 4 °C overnight. The bands were washed and incubated with HRP-labeled Goat Anti-Rabbit IgG (H + L) (A0208, Beyotime Biotechnology, Shanghai, China) for 1 h at room temperature. Then, chemiluminescence reagents (Beyotime Biotechnology, Shanghai, China) were incorporated, and the membranes were exposed by Bio-Rad Laboratory (Bio-Rad, Hercules, CA, USA). Lastly, the protein bands’ gray values were resolved by ImageJ software (fiji-win64 2.16.0). Antibodies against TSG101 (ab125011, Monoclonal) and CD63 (ab134045, Monoclonal) were purchased from Abcam (Cambridge, UK). Antibodies against p-p65 (3033T, Monoclonal) and p-IκBα (2859T, Monoclonal) were purchased from Cell Signaling Technology (Boston, MA, USA). An antibody against GAPDH (A19056, Monoclonal) was purchased from ABclonal (Wuhan, China). Antibodies against P65 (AF1234, Monoclonal) and IκBα (AG2737, Monoclonal) were purchased from Beyotime (Shanghai, China).

### 2.7. qRT-PCR Analysis

The total RNA of the colons was extracted with a Trizol Reagent (Beyotime Biotechnology, Shanghai, China). One μL of total RNA was transformed into cDNA by using the Evo M-MLV RT Kit with gDNA Clean for qPCR II (Accurate Biotechnology, Hunan, China). The mRNA levels of IL-6, IL-10, iNOS, ZO-1, Occludin, Muc2, and β-actin were quantified by the SYBR Green Permix Pro Taq HS qPCR Kit (Accurate Biotechnology, Hunan, China) and the CFX96 Real-Time System (Bio-Rad, Hercules, CA, USA). Primer sequences are listed in Supplementary Table S1. The expression levels were calculated using the 2^−ΔΔCt^ approach and standardized to β-actin expression levels.

### 2.8. Histology and Immunohistochemistry Analysis

The colon tissues were immobilized with 4% paraformaldehyde and embedded in paraffin blocks. The blocks were sectioned into 5 μm slices which were subsequently stained with hematoxylin and eosin. For immunofluorescence analysis, tissue slices were boiled in sodium citrate buffer and incubated in 3% H_2_O_2_. Tissue slices were incubated with 3% BSA at room temperature and then incubated overnight at 4 °C with primary antibodies. The slices were subsequently stained with DAB and hematoxylin after being incubated with secondary antibodies. The sample images were observed using a microscope (RVL-100, ECHO, Singapore) and quantified using Image J software (fiji-win64 2.16.0). 

### 2.9. Microbiota 16S rRNA Gene Sequencing

Fecal DNA was extracted with the TGuide S96 Magnetic Soil/Stool DNA Kit (Tiangen Biotechnology, Beijing, China). NanoDrop (Thermo Fisher, Waltham, USA) was employed to measure samples’ DNA concentrations. The V3-V4 region of 16S rRNA was amplified using forward primer 338F and reverse primer 806R. PCR amplicons were examined on 1.8% agarose electrophoresis, followed by purification and quantification. The library was prepared with NEBNext^®^ Ultra™ DNA LibraryPrep Kit for Illumina^®^ and sequenced on an Illumina Novaseq 6000 (Illumina, San Diego, CA, USA). Sequences were quality-controlled by Trimmomatic v0.33 and denoised by Dada2 (truncated at QIIME2 2020.6), resulting in 59,292 ± 3106 (mean ± s.d.) reads and 548 ± 62 amplicon sequence variants (ASVs) per sample. The taxonomy of the ASVs was annotated based on the SILVA database (Release 138). The alpha diversity indices (observed ASVs, Chao 1 richness, Ace richness, Shannon index, and Simpson index) and beta diversity (binary Jaccard, Bray–Curtis dissimilarity, weighted UniFrac distance, and unweighted UniFrac distance) were calculated based on the ASV profiles. PICRUSt2 was used to estimate microbial functional potentials from the ASV profiles.

### 2.10. Statistical Analysis

All experimental results were presented as means ± standard deviation (SD). Normalized gene expression was expressed as the mean ± the standard error of the mean (SEM). Statistical analyses were conducted by GraphPad Prism (10.2.3, La Jolla, USA). Comparisons of normally distributed data between two groups were made by unpaired Student’s t-test, and among three or more groups by analysis of variance (ANOVA). Linear discriminant analysis effect size (LeEfSe) calculation was performed to detect differentially abundant microbial taxa among groups. Spearman’s correlation tests were performed to analyze correlations between abundances of microbial genera and expression levels of cytokines. *p* values < 0.05 were considered statistically significant, and categorized into the following categories: *p* < 0.05 (*), *p* < 0.01 (**), *p* < 0.001 (***), *p* < 0.0001 (****).

## 3. Results

### 3.1. Identification and Analysis of GMEVs

**Figure 1:**
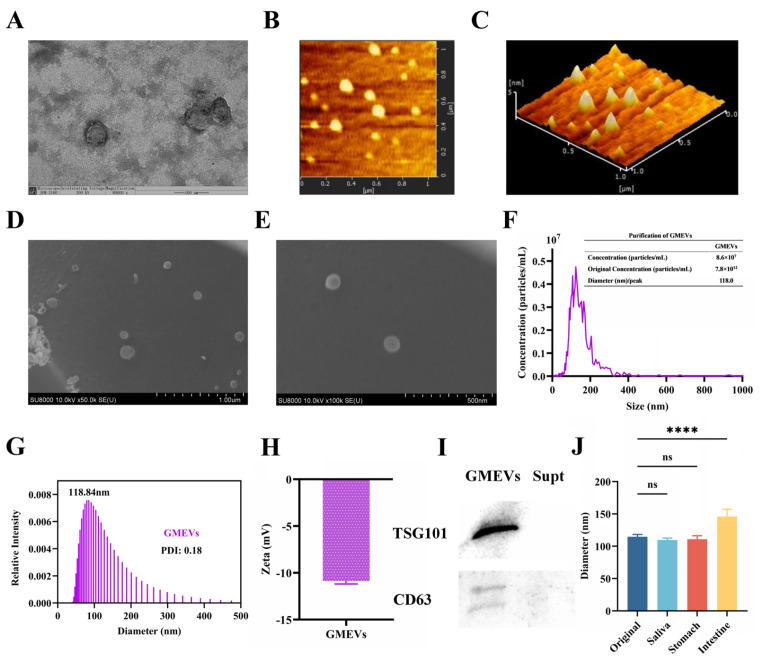
Identification and analysis of goat milk-derived extracellular vesicles (GMEVs). (**A**) TEM image of GMEVs (*n* = 3). Scale bars represent 100 nm. (**B**) AFM image of GMEVs (*n* = 3). (**C**) Three-dimensional rendering of topography image shown in (**B**) (*n* = 3). (**D**,**E**): TEM images of GMEVs (*n* = 3). (**F**) Concentration analysis of GMEVs by NTA (*n* = 3). (**G**) Size distribution of GMEVs analyzed via DLS (*n* = 3). (**H**) Zeta potential of GMEVs (*n* = 3). (**I**) Biomarkers of exosomes by WB (*n* = 3). (**J**) Size distribution of GMEVs before and after going through ex vivo digestion experiment (*n* = 10). **** *p* < 0.0001. ns, no significance.

The GMEVs exhibited an expected, typically cup-shaped morphology, found using transmission electron microscopy (TEM) (Figure 1A). Atomic force microscopy (AFM) showed an inhomogeneous morphology while phase images showed substructures, indicating the existence of variable constitutive elements such as lipids, proteins, and microRNAs (Figure 1B,C). The GMEVs presented a uniform size distribution and a fine circular structure under a scanning electron microscope (SEM) (Figure 1D,E). The nanoparticle tracking analysis (NTA) indicated that the GMEVs contained 7.8 × 10^12^ particles per mL, with a mean size of 118 nm (Figure 1F). The DLS also revealed a mean size of 118 nm (Figure 1G). The zeta potential of the GMEVs, analyzed by Zetasizer Lab, was −10.78 mV, which suggests that the GMEVs were stabilized in PBS (Figure 1H). The marker proteins (CD63, TSG101) of GMEVs were determined by WB (Figure 1I). To examine the oral bioavailability of the GMEVs, we evaluated their stability in artificial saliva, gastric juice, and intestinal fluid with an in vitro digestion experiment. We found no obvious change in the particle sizes of the GMEVs in saliva and gastric juice. However, the particle sizes were increased in intestinal fluid, perhaps indicating the swelling of GMEVs in intestinal fluid (Figure 1J).

### 3.2. GMEVs Relieved DSS-Induced Colitis Symptoms in Mice

**Figure 2:**
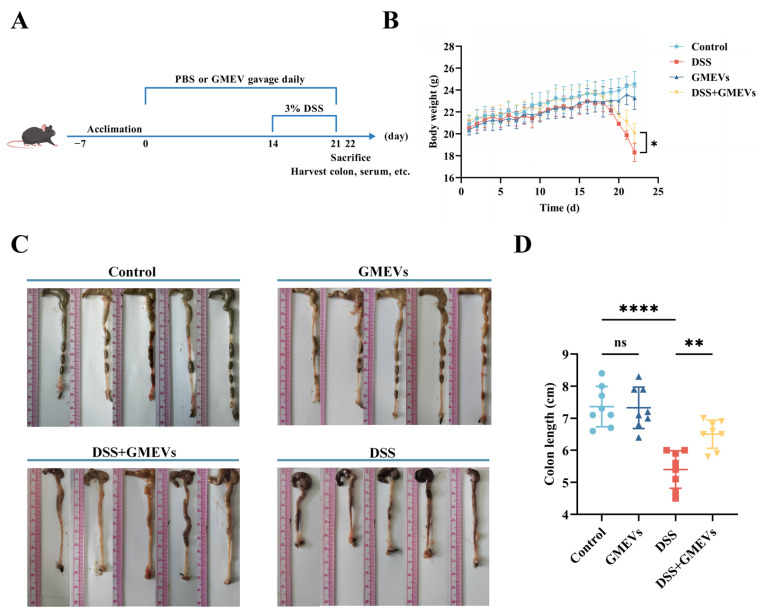
GMEVs relieved dextran sodium sulfate (DSS)-induced colitis symptoms in mice. (**A**) Schematic diagram illustrating administration schedule of GMEVs in DSS-induced mouse model of ulcerative colitis. (**B**) Body weight changes in mice among different treatment groups (*n* = 8). (**C**,**D**): Colon length, determined to assess severity of colonic impairment (*n* = 8). * *p* < 0.05; ** *p* < 0.01; **** *p* < 0.0001. ns, no significance.

Mice were fed following the procedure depicted in Figure 2A; their body weight was measured daily and their colon length was determined at the endpoint (21 days). As expected, the DSS treatment significantly decreased body weight and shortened colon length. These alterations were alleviated with a pre-treatment of GMEVs (DSS + GMEVs). GMEVs alone did not change the body weight or colon length when compared to the control group (Figure 2B–D). These results suggest that GMEV administration could effectively relieve DSS-induced colitis symptoms in mice.

### 3.3. GMEVs Attenuated DSS-Induced Histopathological Changes in Mice

**Figure 3:**
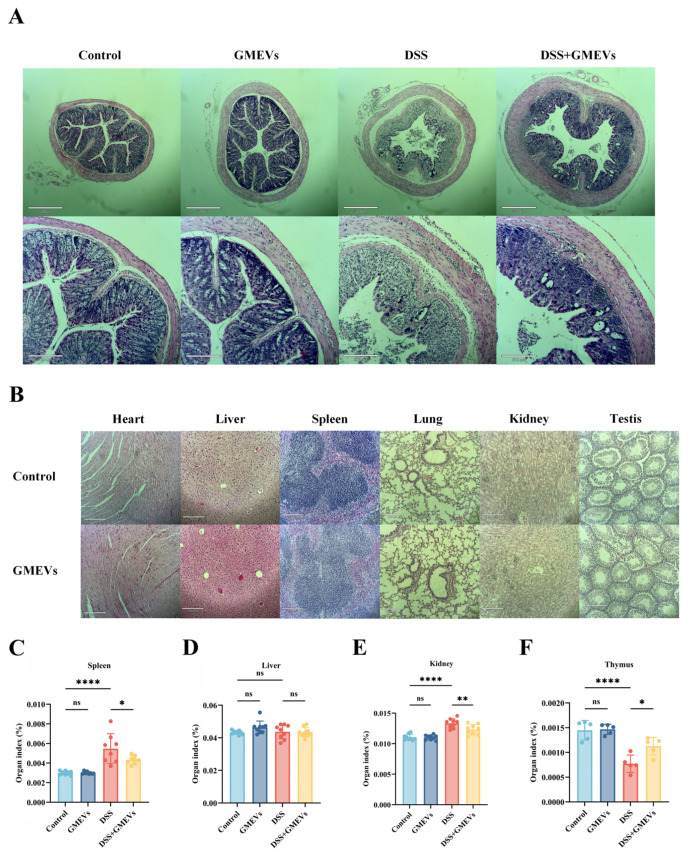
GMEVs attenuated DSS-induced histopathological changes in mice. (**A**) Representative images of H&E-stained colon sections (*n* = 5). (**B**) Histopathological sections of heart, liver, spleen, lung, kidney, and testes from mice in control, DSS, DSS + GMEVs, and GMEVs groups (*n* = 5). Scale bars: 210 μm. (**C**–**F**) Organ index of main organs in four groups of mice. * *p* < 0.05; ** *p* < 0.01; **** *p* < 0.0001. ns, no significance.

With reference to the normal morphology and structure of colon tissues in the control group, we found no histological lesions in the GMEVs group. Echoing previous studies, the DSS treatment induced serious colitis symptoms, including mucosal injury, epithelial cell decay and erosion, extensive goblet cell depletion and crypt loss, and inflammatory cell infiltration [30]. Notably, the GMEV pre-treatment dramatically alleviated these DSS-induced damages (Figure 3A).

In order to evaluate the oral biological safety of GMEVs in mice, we analyzed histopathological sections of the main organs. We did not observe rhabdomyolysis, necrosis, or apoptosis in the heart, nor fatty degeneration, necrosis, or edema degeneration in the liver. The tissue structures of the spleen and lung were intact. The glomeruli, renal tubules, and renal interstitium were clearly distinguishable in the kidney. In the testes, the seminiferous tubules had intact basement membranes, regular lumens, and spermatogenic epithelium (Figure 3B–F). Together, these results show that the GMEVs were effective and safe.

### 3.4. GMEVs Improved DSS-Induced Intestinal Barrier Damage

**Figure 4:**
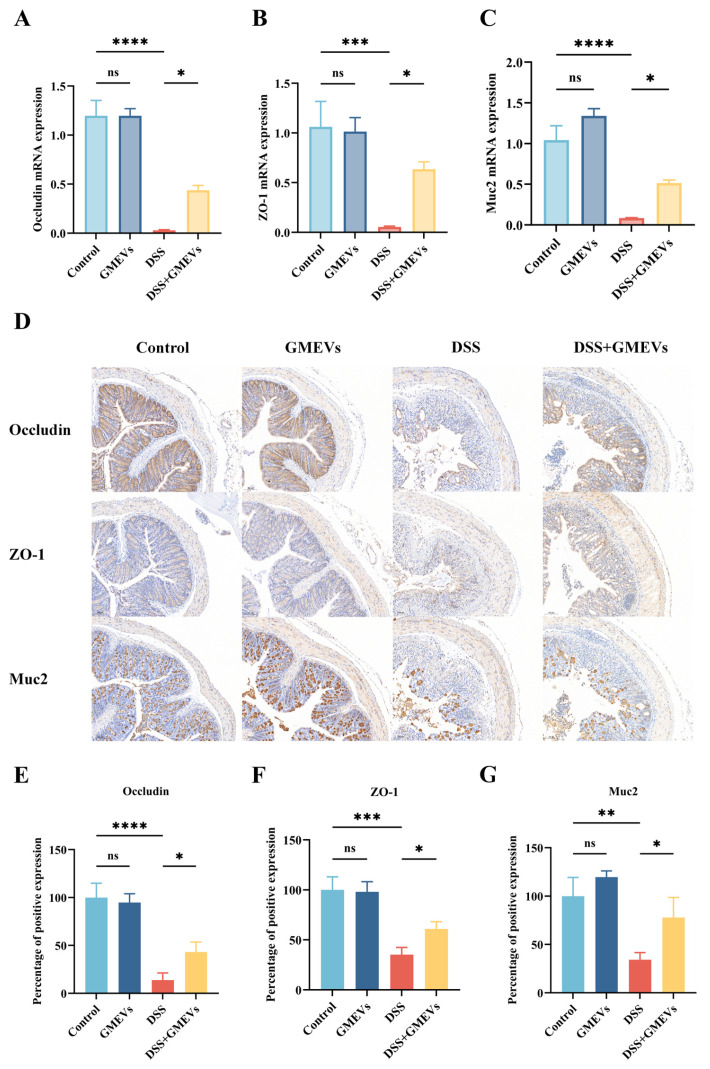
GMEVs improved DSS-induced intestinal barrier dysbiosis. (**A**–**C**) The mRNA expression levels of Occludin, ZO-1, and Muc2 in the colons (*n* = 5). β-actin was used as the reference gene. (**D**–**G**) The quantification and staining of Occludin, ZO-1, and Muc2 in colon tissues through immunohistochemistry in the four groups of mice (*n* = 3). The scale bars indicate 100 μm. * *p* < 0.05, ** *p* < 0.01, *** *p* < 0.001, **** *p* < 0.0001. ns, no significance.

The intestinal epithelium serves as a barrier that prevents pro-inflammatory factors (such as toxins, antigens, and pathogens) from infiltrating into mucosal tissues and the circulatory system [31,32,33]. The intestinal barrier is mostly modulated by the apical junctional complexes, composed of adherent junctions and tight junctions (TJs) [34]. Here, we evaluated the expression levels of TJs (ZO-1 and Occludin) and colonic mucin 2 (Muc2) in order to test the protective role of GMEVs against DSS-induced intestinal barrier damage. DSS treatment significantly reduced the expression of zonula occludens-1 (ZO-1), Occludin, and Muc2, which were restored with GMEVs administration. In addition, no differential expressions of these genes were observed between the control group and the GMEVs group (Figure 4A–C).

The protein levels of TJs and Muc2 in the colon were further analyzed by immunohistochemistry. Consistent with the mRNA expression levels, after DSS stimulation, the protein expressions of ZO-1, Occludin, and Muc2 conspicuously reduced, whereas pre-treatment with GMEVs partially reinstated the expressions of these proteins in DSS-induced mice. Again, GMEVs did not induce intestinal barrier damage when compared to the control group (Figure 4D–G). The above results show that GMEVs alone did not affect the secretion of TJs and Muc2 in normal mice, and that they can improve the intestinal barrier in DSS-induced colitis.

### 3.5. GMEVs Modulated Inflammatory Cytokine Secretion

**Figure 5:**
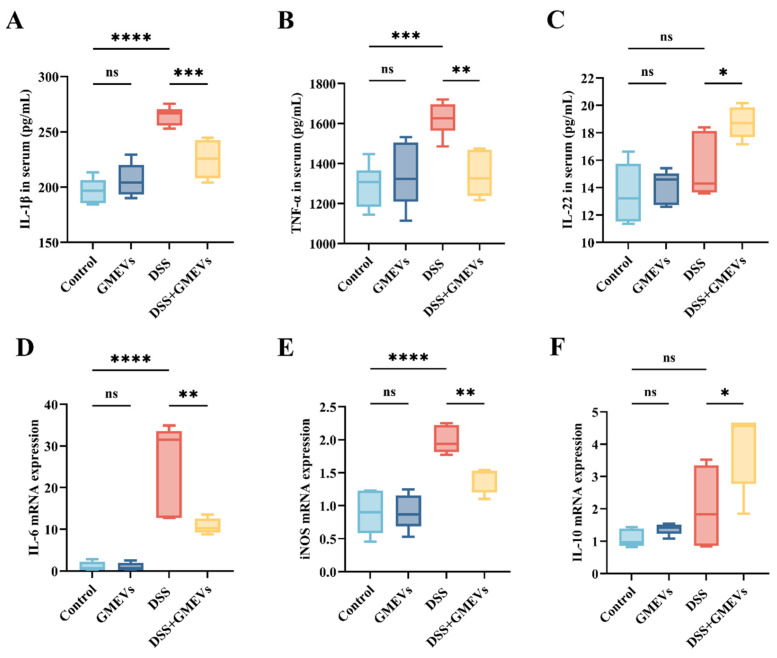
GMEVs modulated inflammatory cytokine secretion. (**A**) IL-1β (*n* = 6), (**B**) TNF-α (*n* = 6), (**C**) IL-22 (*n* = 5), (**D**) IL-6 (*n* = 5), (**E**) iNOS (*n* = 5), and (**F**) IL-10 (*n* = 5). * *p* < 0.05; ** *p* < 0.01; *** *p* < 0.001; **** *p* < 0.0001. ns, no significance.

Interleukin-1 beta (IL-1β), secreted by activated macrophages and intestinal epithelial cells, exacerbates mucosal ulceration by activating the nuclear factor kappa B (NF-κB) signaling pathway to promote the release of pro-inflammatory cytokines such as tumor necrosis factor-α (TNF-α) and interleukin-6 (IL-6) [35]. TNF-α not only directly induces apoptosis in intestinal epithelial cells but also amplifies the inflammatory cascade by enhancing vascular permeability and adhesion molecule expression [36]. IL-6, being the signal transducer, drives immune imbalance and chronic inflammation by activating the signal transducer and activator of transcription 3 (STAT3) signaling pathway, which promotes Th17 cell differentiation while suppressing regulatory Treg cell function [37]. The overexpression of inducible nitric oxide synthase (iNOS) generates excessive nitric oxide (NO), causing deoxyribonucleic acid (DNA) damage and mitochondrial dysfunction through nitrosative stress, thereby further aggravating tissue injury [38]. These molecules synergistically form a vicious cycle that drives the pathological progression of UC from acute inflammation to chronic and irreversible tissue damage. Both being the most typical cytokines in the interleukin-10 (IL-10) family, IL-10 is considered the most crucial anti-inflammatory cytokine, while interleukin-22 (IL-22) is thought to specifically act on epithelial cells, promoting cell regeneration and tissue repair [39,40]. According to existing literature, upregulations of IL-10 and IL-22 expression levels could alleviate intestinal inflammation [41,42]. To characterize the inflammatory reactions triggered by DSS-induced intestinal barrier damage, we examined the expression levels of six selected cytokines (IL-1β, TNF-α, IL-6, iNOS, IL-10, and IL-22) in the serum and colon using ELISA and qRT-PCR. The expressions of the pro-inflammatory cytokines IL-1β, IL-6, TNF-α, and iNOS were elevated in the serum and colon of the DSS group, the magnitudes of which were significantly reduced with the GMEVs pre-treatment (Figure 5A,B,D,E). In addition, the expression levels of the anti-inflammatory IL-10 and IL-22 in the DSS + GMEVs group were considerably greater than in the DSS group, with this difference perhaps underlying the beneficial effect of GMEV pre-treatment for relieving colitis (Figure 5C,F). There were no differences in the six cytokines in the colon and serum between the control and GMEVs groups, which proved that the GMEVs had no effect on inflammatory cytokines in normal mice. These results prompted further investigation into the underlying mechanism by which GMEVs could modulate inflammatory cytokine secretion to protect mice from colitis.

### 3.6. GMEVs Ameliorated Colitis by Modulating the NF-κB Signaling Pathway

**Figure 6:**
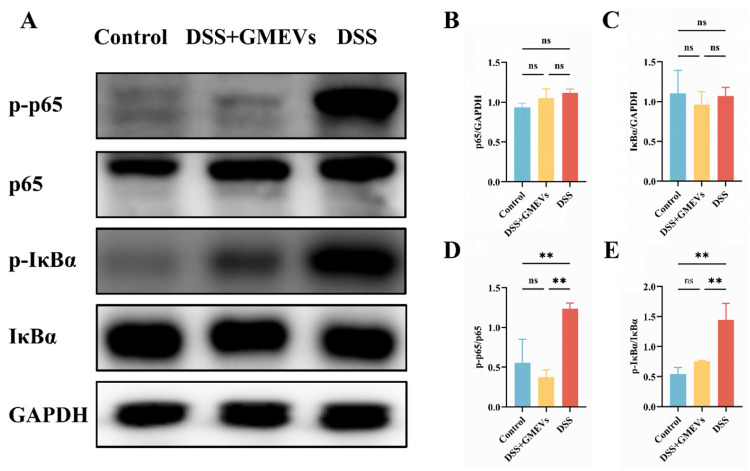
GMEVs ameliorated colitis by modulating the NF-κB signaling pathway. (**A**) Representative WBs of p-p65, p65, p-IκBα, IκBα, and GAPDH in the colons (*n* = 3). (**B**–**E**) The quantification of the protein expression levels of p-p65, p65, p-IκBα and IκBα, normalized to GAPDH (*n* = 3). ** *p* < 0.01. ns, no significance.

Numerous studies have evidenced that the NF-κB signaling pathway plays an essential role in the pathogenic development of UC [43,44]. Therefore, we detected the expressions of several key regulatory factors in the NF-κB signaling pathway via WB (Figure 6). We observed no significant difference in the protein expressions of p65 and IκBα between the DSS and DSS + GMEVs groups (Figure 6A–C). The protein expressions of p-p65 and p-IκBα were clearly higher in the DSS group than those in the control group. However, GMEV administration effectively suppressed these alterations when compared to the DSS group (Figure 6A,D,E). Our findings confirm that GMEVs ameliorated DSS-induced colitis by modulating the NF-κB signaling pathway.

### 3.7. GMEVs Regulated Gut Microbiota Dysbiosis in DSS-Induced Colitis Mice

**Figure 7:**
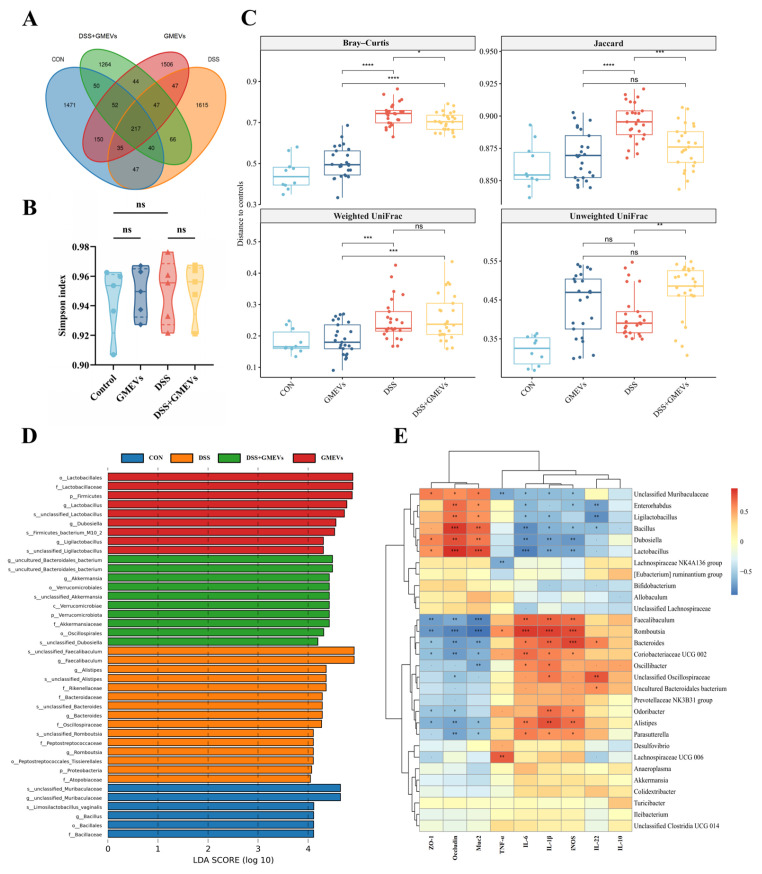
GMEVs regulated gut microbiota dysbiosis in DSS-induced colitis mice. (**A**) Venn diagram of amplicon sequence variants in stool samples (*n* = 5). (**B**) Simpson index (*n* = 5). (**C**) Beta diversity (*n* = 5). (**D**) LDA indicated most differential abundant bacterial taxa in each group of mice (*n* = 5). (**E**) Correlation analysis of specific gut microbiota, intestinal barrier-related genes, and inflammatory cytokines between four groups (*n* = 5). Color represents Spearman’s correlation coefficient (Rho), FDR-corrected *p* values denoted as follows: *p* < 0.1; * *p* < 0.05; ** *p* < 0.01; *** *p* < 0.001; **** *p* < 0.0001. ns, no significance.

Since gut microbiota dysbiosis is implicated in DSS-induced colitis [45,46], we further investigated the impact of GMEVs on the interaction between gut microbiota and the intestinal barrier in DSS-induced colitis. We found that there were 217 overlapping ASVs among the four groups, with 66 overlapping ASVs between the DSS and DSS + GMEVs groups (Figure 7A). There was no significant difference in the alpha diversity among the four groups (Figure 7B and Figure S1). As seen in Figure 7C, for Bray–Curtis and Jaccard dissimilarity, the DSS + GMEVs group was closer to the controls than the DSS group, indicating that GMEV supplementation may somewhat protect the gut microbiota from DSS exposure.

At the phylum level, the relative abundance of *Proteobacteria* was significantly increased in the DSS group (*p* < 0.01), but not in the DSS + GMEVs group (*p* > 0.05) (Figure S2), perhaps explained by its class, *Gammaproteobacteria* (Figure S3A). At the order level, the relative abundance of *Burkholderiales* was significantly increased (*p* < 0.05), and *Lactobacillales* was significantly decreased in the DSS group (*p* < 0.05), while GMEVs remarkably reversed their abundances, as seen in the DSS + GMEVs group (Figure S3B). At the family level, the DSS group displayed an increase in *Rikenellaceae* (*p* < 0.05), *Marinifilaceae* (*p* < 0.05), *Peptostreptococcaceae* (*p* < 0.001), and *Sutterellaceae* (*p* < 0.01), which were significantly decreased through GMEV administration in the DSS + GMEVs group. In addition, the relative abundances of *Muribaculaceae* (*p* < 0.001) and *Lactobacillaceae* (*p* < 0.05) in the DSS group were lower than those in the DSS + GMEVs group (Figure S3C). At the genus level, the relative abundances of an *unclassified Muribaculaceae* (*p* < 0.01), *Dubosiella* (*p* < 0.001), and *Lactobacillus* (*p* < 0.05) were significantly decreased, while *Alistipes* (*p* < 0.01), *Odoribacter* (*p* < 0.05), *Romboutsia* (*p* < 0.001), *Parasutterella* (*p* < 0.01), and *Lachnospiraceae_UCG_006* (*p* < 0.05) were significantly increased in the DSS group. However, GMEV administration notably reversed these changes (Figure S3D). These findings were further supported by the LDA analysis (Figure 7D and Figure S4).

In addition, metabolic pathway profiles of the microbiota, including the Clusters of Orthologous Genes (COGs) and the Kyoto Encyclopedia of Genes and Genomes (KEGG) pathways, were predicted by PICRUSt2 and summarized by contributing phyla and genera. The results show that Proteobacteria contributes more to functions relevant to substance dependence, neurodegenerative diseases, infection, and drug resistance compared to other functions (Figure S5A,B). Likewise, *Romboutsia* has excessive contributions to functions relevant to infectious diseases, neurodegenerative diseases, and immune diseases compared to its contributions to other functions (Figure S5C,D). The *unclassified Lachnospiraceae* and *unclassified Muribaculaceae* were the top two contributors to the relative abundances of the functions, contributing on average 17.8% and 14.8% of the KEGG functions and 18.9% and 10.7% of the COG functions, respectively (Figure S5E,F).

Finally, our correlation analysis revealed potential interactions among gut microbiota, intestinal barrier-related genes, and inflammatory cytokines (Figure 7E). For example, increases in harmful bacteria such as *Romboutsia*, *Alistipes*, *Lachnospiraceae_UCG_006*, and *Parasutterella* were positively correlated with the expression of pro-inflammatory cytokines but negatively correlated with the expression of intestinal barrier-related genes. In contrast, abundances of indigenous bacteria such as the *unclassified Muribaculaceae*, *Lactobacillus*, and *Dubosiella* were negatively correlated with the expression of pro-inflammatory cytokines and positively correlated with the expression of intestinal barrier-related genes.

## 4. Discussion

The intestine is the primary place to digest and absorb nutrients, and the intestinal barrier is crucial to preserving the body’s health [47]. Harmful microorganisms and toxicants are able to access the intestine and damage the intestinal barrier when intestinal immunity is compromised [48]. The mucosal barrier serves as the first barrier of the intestine, which prevents intestinal bacteria and colon epithelial cells from coming into direct contact [49]. According to a previous report, milk-derived EVs could safeguard against necrotizing enterocolitis by regulating Muc2 expression [50]. The intestinal epithelial cell barrier is sustained by TJs, such as Occludin, Claudins, and ZO-1 [51]. These TJs seal the paracellular gap between epithelial cells and adjust the access of water, ions, and nutrients while limiting the invasion of pathogens, thereby modulating the barrier function of epithelial cells [52]. Tong and others found that bovine and human milk-derived EVs could upregulate the expression levels of Muc2, ZO-1, and Occludin to improve intestinal barrier function and reduce colitis in mice [53]. In this study, we showed that GMEVs also enhanced the secretion of Muc2, ZO-1, and Occludin that reinforced the intestinal barrier.

Under pathological conditions where the intestinal barrier is damaged, a mass of pro-inflammatory cytokines are secreted into the colon tissue and serum. Our findings showed that GMEV administration significantly alleviated DSS-induced increases in IL-1β, IL-6, TNF-α, and iNOS, while elevating IL-10 and IL-22. One of the classical signaling mechanisms to adjust inflammatory response implicated in UC is the NF-κB signaling pathway, which is responsible for regulating the transcription of inflammatory genes [54]. Upon activation, NF-κB promotes a massive release of pro-inflammatory cytokines that trigger inflammation cascades, thus mediating the intestinal mucosal immune response in UC [35,55]. In this research, we revealed that the application of GMEVs could reduce the secretion of pro-inflammatory cytokines and increase the expression of anti-inflammatory cytokines by inhibiting IκBα and NF-κB p65 phosphorylation in the NF-κB signaling pathway, improving the symptoms of DSS-induced colitis. These data clearly indicate that NF-κB inactivation is, at least in part, the possible mechanism by which GMEVs decrease the susceptibility of mice to DSS-induced colitis.

The gut microbiota plays a vital role in the development of colitis [56]. Various immunological mediators, including cytokines and chemokines, secreted from intestinal epithelial cells and stimulated by gut microbiota, can modulate host immune responses, maintaining a well-balanced relationship between gut microbes and the host immune system [57]. As previously reported, colitis induced by DSS led to gut microbiota dysbiosis, including a decline in the diversity of gut microbiota and a rise in pathogenic bacteria [45]. Previous studies had already proved that milk-derived EVs could change the composition of gut microbiota [58,59]. Here, we showed that DSS exposure elevated the relative abundances of *Proteobacteria*, *Gammaproteobacteria*, *Burkholderiales*, *Rikenellaceae*, *Marinifilaceae*, *Peptostreptococcaceae*, and *Sutterellaceae*, and that GMEV administration could prevent these elevations. The result predicted by PICRUSt2 indicated that an increase in Proteobacteria may predispose the mice to inflammation. These findings were consistent with previous studies showing positive associations between these taxa and the severity of DSS-induced colitis in mice [60,61,62,63,64,65,66]. Our results also show that the relative abundance of *Parasutterella* was significantly increased in the DSS group, while the abundance was significantly decreased with GMEV supplementation. Interestingly, Ibrahim et al. have shown that *Parasutterella* was enriched after AOM/DSS treatment, which led to gut barrier dysbiosis and chronic inflammation, suggesting that *Parasutterella* may be a potentially harmful bacterium [67]. Our data also showed that GMEVs increase the relative abundance of *Lactobacillus*. In mice, the oral administration of *Lactobacillus*-secreted IL-10 reduced the incidence of DSS-induced colitis by 50% [68]. In addition, compared to the control group, DSS exposure reduced the relative abundance of *Dubosiella*, which was not seen with GMEVs pre-treatment. This indicates that *Dubosiella* may be potentially beneficial in the fight against UC in mice, as previously reported [69].

Finally, we established potential correlations among gut microbiota, expressions of intestinal barrier-related genes, and inflammatory cytokines. Several studies had reported that *Muribaculaceae* was negatively correlated with colitis and had a strong correlation with short-chain fatty acid (SCFA) metabolism pathways [70,71,72]. Therefore, this indigenous microbe may help produce SCFAs and regulate intestinal barrier functions to maintain gut homeostasis. Our results showed that depletion of *unclassified Muribaculaceae* may lead to functional dysbiosis in the microbiota, and GMEV treatment prevented the reduction in *unclassified Muribaculaceae* seen in the DSS group. However, whether the GMEVs specifically target the regulation of gut microbiota metabolites to affect intestinal homeostasis needs to be further verified. Research on gut microbiota metabolites, particularly SCFAs, is critical for understanding host–microbe interactions and advancing disease prevention and treatment. As core products of dietary fiber metabolized by gut microbiota, SCFAs are directly linked to the development of chronic diseases such as obesity, diabetes, and inflammatory bowel disease through their roles in maintaining intestinal barrier function, regulating immune responses, and modulating metabolism [73,74]. In recent years, an effective approach for modulating gut microbiota, fecal microbiota transplantation (FMT) has further elucidated the causal relationships between microbiota, SCFAs, and disease [75]. For example, FMT from multiple donors into recipients with active UC significantly restores SCFA levels, thereby improving intestinal barrier integrity, suppressing excessive inflammation, and alleviating metabolic dysregulation [76]. Furthermore, fluctuations in SCFA concentrations can serve as diagnostic biomarkers for diseases. For instance, inflammatory bowel disease patients exhibit markedly reduced fecal butyrate levels, while the FMT-mediated restoration of microbial balance correlates with butyrate recovery and clinical symptom improvement [77]. Therefore, the targeted modulation of gut microbiota and their metabolites through dietary interventions, probiotics, or FMT has emerged as a potential therapeutic strategy. Despite challenges such as validating causality, navigating the complexity of metabolic networks, and optimizing delivery technologies, this field offers a pivotal breakthrough for unraveling microbe–host coevolution mechanisms and developing novel approaches to prevent and manage chronic diseases. Therefore, whether GMEVs affect colitis by regulating the metabolites of gut microbiota needs further investigation.

This study has several limitations. First, while the preventive value of GMEVs in DSS-induced colitis is established in this study, their therapeutic potential for treating colitis needs to be further examined. Second, the long-term impact of GMEVs on host physiology, as well as the durability of their protective effect on DSS-induced colitis, should be investigated with longer follow-ups. In addition, although we characterized the effects of GMEVs through multiple lenses, only a select panel of genes and proteins were analyzed. Future studies with multi-omics measurements (e.g., transcriptomics, proteomics, and metabolomics) are warranted to comprehensively depict the systems interactions and to identify the functional components (e.g., microRNAs or proteins) of the GMEVs. Similarly, profiling the microbiota with whole-genome shotgun sequencing will help identify key microbial players at a higher resolution. Furthermore, the proposed mechanisms should be further validated, for example, in NF-κB knock-out/deficient mice. Lastly, these findings were derived from a mouse model of DSS-induced colitis, and their translational value into human UC deserves further investigation.

## 5. Conclusions

This study confirmed that the oral administration of 20 mg/kg/d of GMEVs effectively alleviated weight loss, colon shortening, and organ damage in DSS-induced colitis mice while demonstrating good oral biosafety. Furthermore, GMEV pre-treatment upregulated the expression of ZO-1, Occludin, and Muc2, promoted the secretion of anti-inflammatory cytokines IL-10 and IL-22, inhibited the release of pro-inflammatory cytokines IL-1β, IL-6, TNF-α, and iNOS, and blocked the abnormal activation of the NF-κB signaling pathway, thereby mitigating the inflammatory response. Notably, GMEV intervention reshaped the gut microbiota in colitis mice, improving disease symptoms. In summary, we demonstrated that the oral administration of GMEVs could alleviate intestinal inflammation by modulating the gut microbiota in DSS-induced colitis. Therefore, dietary formulations supplemented with GMEVs may have a great potential to protect and maintain intestinal health. Further research is needed to identify the functional constituents of GMEVs, to clarify their mechanisms of action in colitis, and to demonstrate their long-term effects. As natural nanoscale vesicles, GMEVs demonstrate multifaceted potential in the fields of functional foods and medical therapeutics. Enriched with bioactive components such as functional proteins, lipids, and nucleic acids, these vesicles exert health intervention effects through mechanisms including immune response modulation, inflammation suppression, and cellular repair promotion. They exhibit particular clinical translational potential in addressing gastrointestinal disorders, chronic inflammatory conditions, and metabolic syndrome. On the industrial level, goat milk, as a raw material that can be easily scaled up, combined with optimized exosome isolation and purification techniques, can facilitate the development of low-cost and highly stable functional additives or drug delivery carriers. Additionally, the natural origin and potential low immunogenicity of GMEVs provide novel pathways for creating better-tolerated nutritional supplements or targeted therapeutic formulations. Bridging food safety with clinical demands, GMEVs may emerge as an innovative direction in the interdisciplinary health industry with both commercial value and scientific significance.

## Data Availability

The data presented in this study are available on request from the corresponding author (accurately indicate status).

## Supplementary Materials

The following supporting information can be downloaded at: https://www.mdpi.com/article/10.3390/foods14091514/s1, Figure S1: Alpha diversity for microbiota 16S rRNA gene sequencing; Figure S2: Relative abundances of bacterial phyla across groups; Figure S3: Relative abundances of bacterial classes, orders, families and genera across groups; Figure S4: Cladogram showing differentially abundance taxa among groups based on the LeEfSe analysis; Figure S5: Predicted COG/KEGG metabolic pathway profiles; Table S1: Primer sequences for qRT-PCR analysis.
