# Supplementary material for: Goat Milk-Derived Extracellular Vesicles Alleviate Colitis Potentially Through Improved Gut Microbiota in Mice
Source: Foods. 2025 Apr 26;14(9):1514. doi: 10.3390/foods14091514 (PMC12071645; doi:10.3390/foods14091514)
Supplement: Supplementary file 1 [file foods-14-01514-s001.zip › foods-3531277-supplementary.pdf]

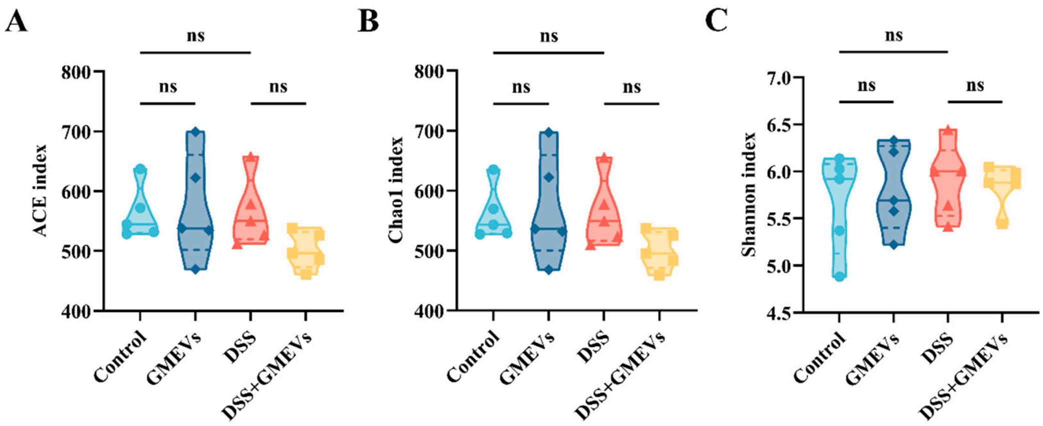

Figure S1. Alpha diversity for microbiota 16S rRNA gene sequencing (n=5). Comparisons were made by Student's t-test.

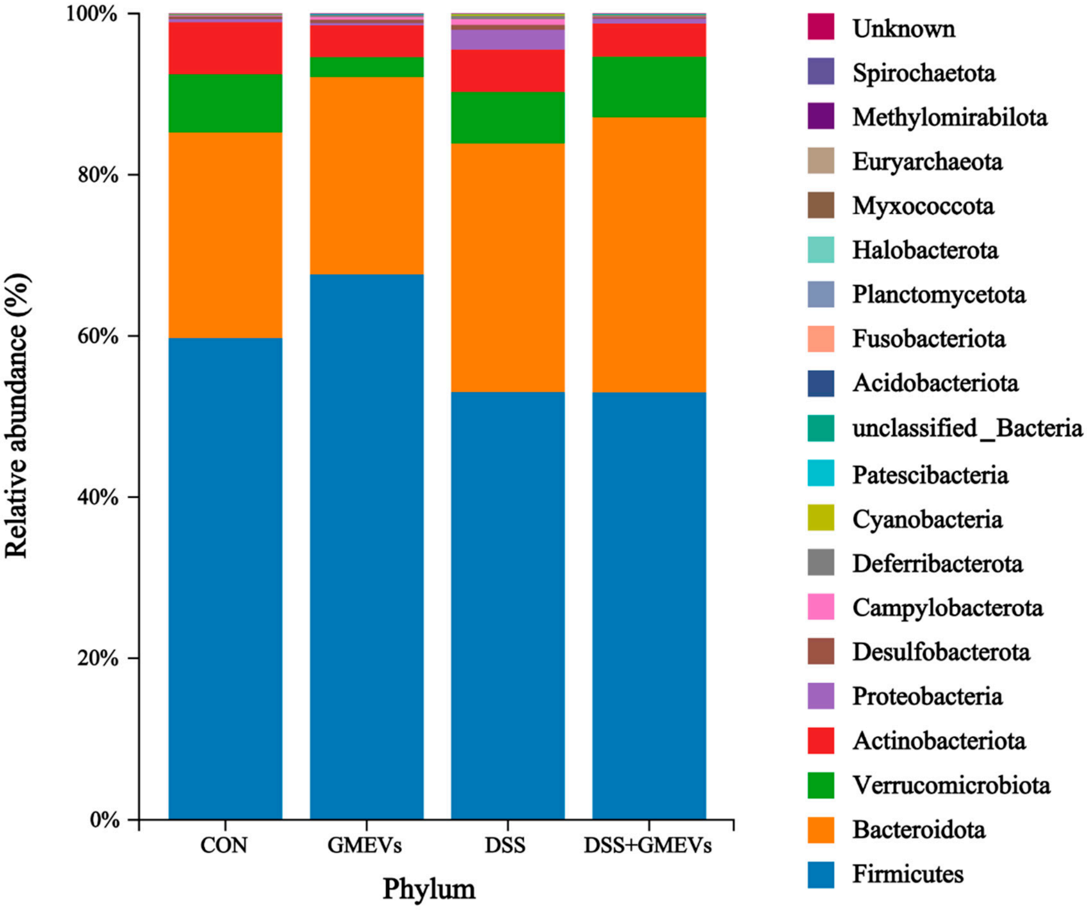

Figure S2. Relative abundances of bacterial phyla across groups (n=5).

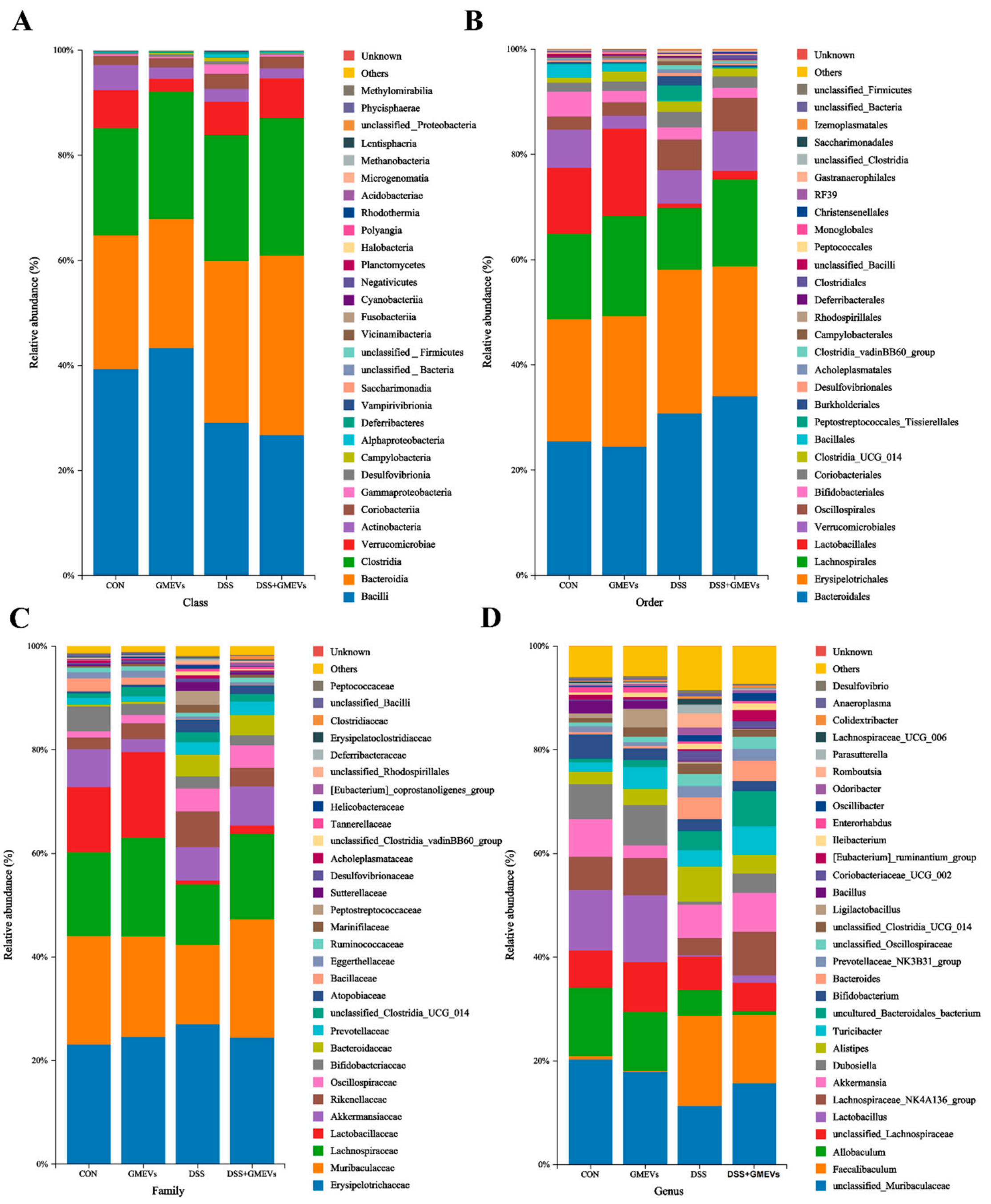

Figure S3. Relative abundances of bacterial classes (A), orders (B), families (C) and genera (D) across groups (n=5).

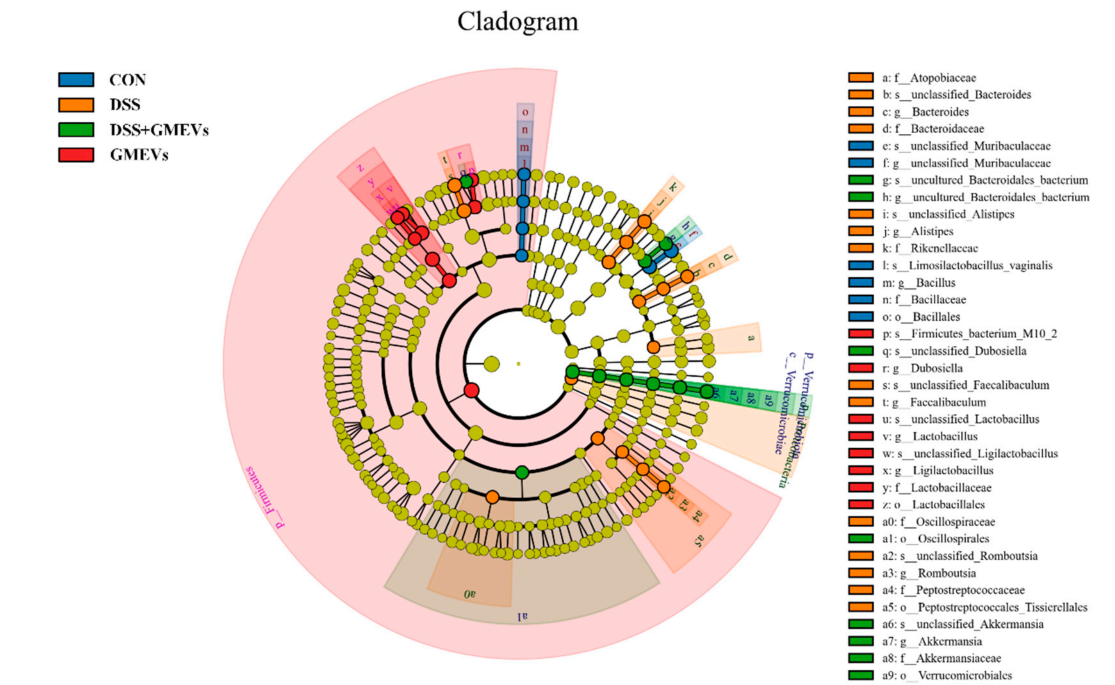

Figure S4. Cladogram showing differentially abundance taxa among groups based on the LeEfSe analysis (n=5).

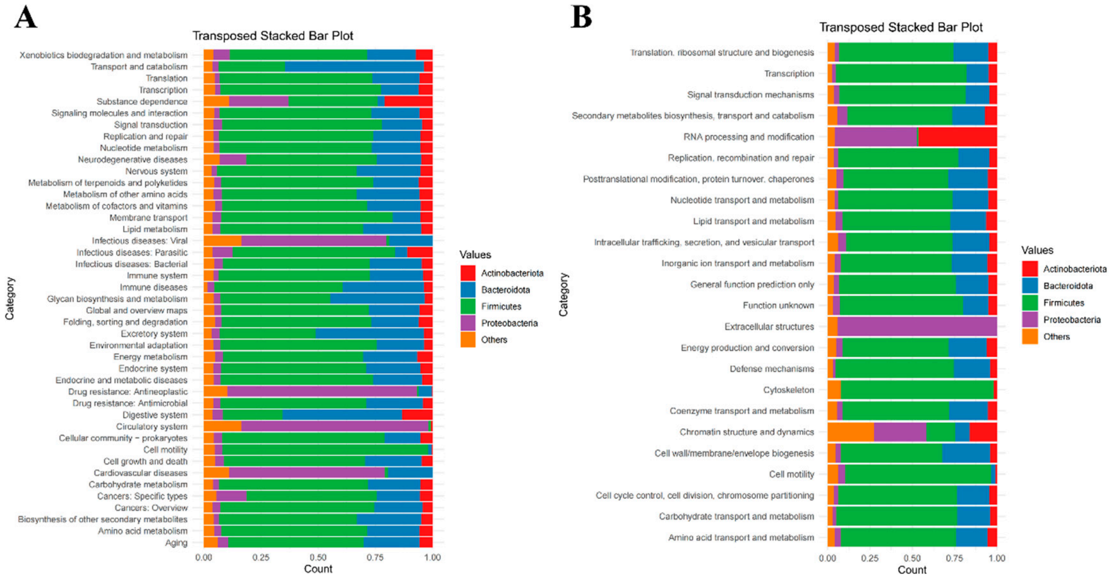

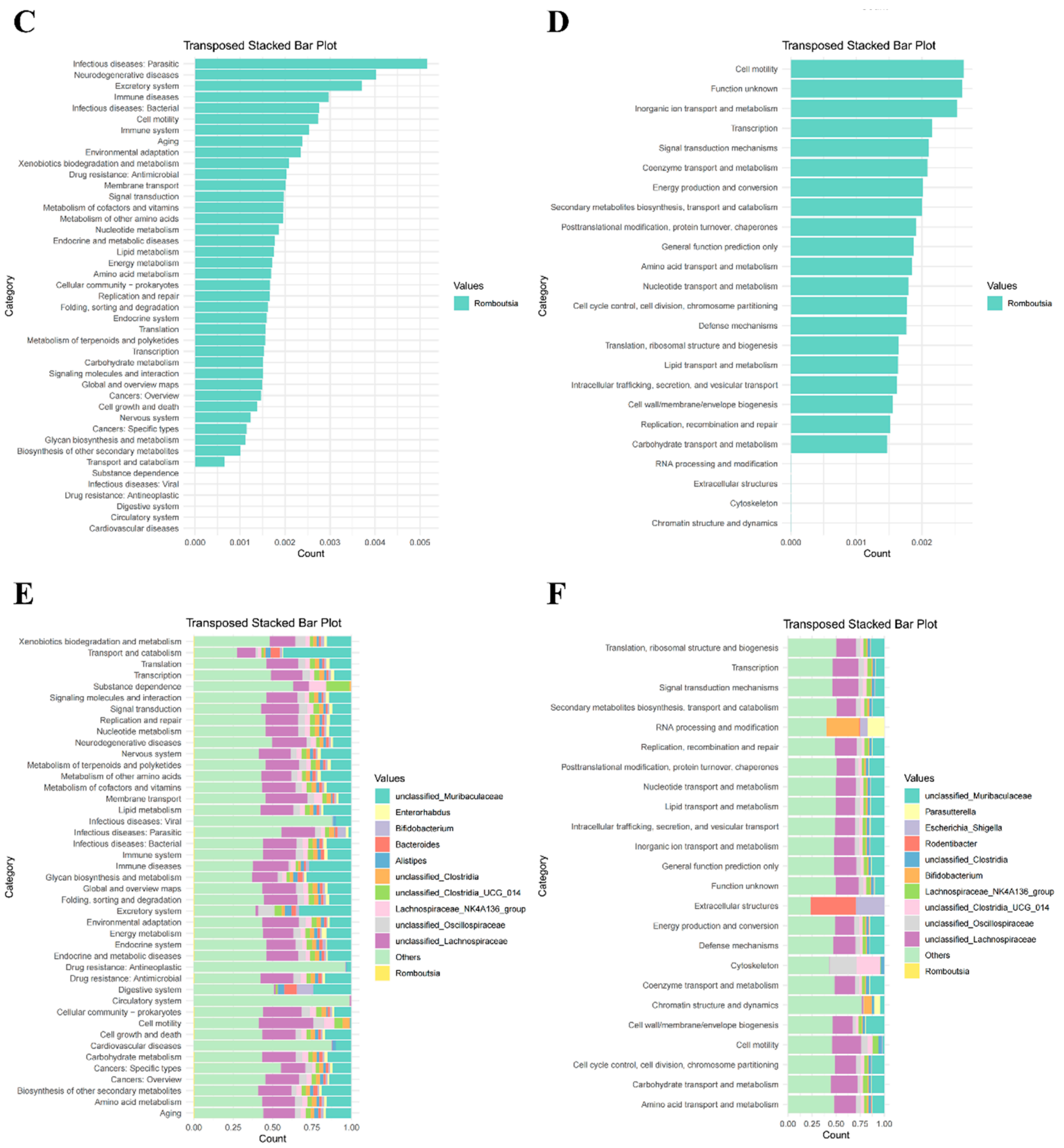

Figure S5. Predicted COG/KEGG metabolic pathway profiles (n=5). (A) KEGG metabolic pathway at phyla, (B) COG metabolic pathway at phyla, (C) KEGG metabolic pathway at *Romboutsia*, (D) COG metabolic pathway at *Romboutsia*, (E) KEGG metabolic pathway at genera, (F) COG metabolic pathway at genera.

Table S1. Primer sequences for qRT-PCR analysis.

Gene	Forward Primer Sequence	Reverse Primer Sequence
IL-6	TGGAAATGAGAAAAGAGTTGTGC	CCAGTTTGGTAGCATCCATCA
IL-10	TACTCGGCAAACCTAGTGCG	GTGTCCCAACATTCATAATTGTCAGT
iNOS	ATGTCCGAAGCAAACATCAC	TAATGTCCAGGAAGTAGGTG
ZO-1	GACCAATAGCTGATGTTGCCAGAG	TATGAAGGCGAATGATGCCAGA
Occludin	GGCAAGCGATCATACCCAGAG	AGGCTGCCTGAAGTCATCCAC
Muc2	GGTCCAGGGTCTGGATCACA	GCTCAGCTCACTGCCATCTG
β-actin	TCAGCAAGCAGGAGTACGATG	AACGCAGCTCAGTAACAGTCC
